# Mass Spectrometry-Based Proteomics for Pre-Eclampsia and Preterm Birth

**DOI:** 10.3390/ijms160510952

**Published:** 2015-05-14

**Authors:** Kai P. Law, Ting-Li Han, Chao Tong, Philip N. Baker

**Affiliations:** 1 China-Canada-New Zealand Joint Laboratory of Maternal and Fetal Medicine, Chongqing Medical University, Chongqing 400016, China; E-Mails: t.han@auckland.ac.nz (T.-L.H.); philip.baker@auckland.ac.nz (P.N.B.); 2 The Liggins Institute, University of Auckland, Auckland 1142, New Zealand; 3 Department of Obstetrics, the First Affiliated Hospital of Chongqing Medical University, Chongqing 400016, China

**Keywords:** pre-eclampsia, preterm birth, mass spectrometry, proteomics, protein biomarkers

## Abstract

Pregnancy-related complications such as pre-eclampsia and preterm birth now represent a notable burden of adverse health. Pre-eclampsia is a hypertensive disorder unique to pregnancy. It is an important cause of maternal death worldwide and a leading cause of fetal growth restriction and iatrogenic prematurity. Fifteen million infants are born preterm each year globally, but more than one million of those do not survive their first month of life. Currently there are no predictive tests available for diagnosis of these pregnancy-related complications and the biological mechanisms of the diseases have not been fully elucidated. Mass spectrometry-based proteomics have all the necessary attributes to provide the needed breakthrough in understanding the pathophysiology of complex human diseases thorough the discovery of biomarkers. The mass spectrometry methodologies employed in the studies for pregnancy-related complications are evaluated in this article. Top-down proteomic and peptidomic profiling by laser mass spectrometry, liquid chromatography or capillary electrophoresis coupled to mass spectrometry, and bottom-up quantitative proteomics and targeted proteomics by liquid chromatography mass spectrometry have been applied to elucidate protein biomarkers and biological mechanism of pregnancy-related complications. The proteomes of serum, urine, amniotic fluid, cervical-vaginal fluid, placental tissue, and cytotrophoblastic cells have all been investigated. Numerous biomarkers or biomarker candidates that could distinguish complicated pregnancies from healthy controls have been proposed. Nevertheless, questions as to the clinically utility and the capacity to elucidate the pathogenesis of the pre-eclampsia and preterm birth remain to be answered.

## 1. Introduction

Most pregnant women experience minor discomforts during pregnancy, but for a minority of women, serious complications may occur. These pregnancy complications range from ectopic pregnancy, miscarriage, placenta praevia and placenta abruption, and pre-eclampsia, *etc.* In the worst-case scenarios, these conditions may lead to morbidity, disability, and maternal or fetal deaths.

Hypertensive disorders of pregnancy affect around 10% of all pregnancies globally [1]. Pre-eclampsia is the most conspicuous among pregnancy-related hypertensive disorders for its impact on maternal and neonatal health. Other hypertensive disorders occur during pregnancy (e.g., gestational hypertension and chronic hypertension) are usually not as acute or life-threatening. Pre-eclampsia, one of the leading causes of maternal and perinatal mortality and morbidity worldwide, affects 2%–5% of all pregnancies [2]. Pre-eclampsia is characterized by hypertension (≥140/90 mmHg) and proteinuria (≥300 mg in a 24-h urine) and its signs usually only manifest at the last trimester of pregnancy. The condition is often associated with eclampsia and the HELLP syndrome, both of which are potentially life-threatening serious complications. Eclampsia is characterized by grand mal-like seizures, whereas the HELLP syndrome manifests itself with hemolysis, elevated liver enzymes, and low platelet counts. The progression of the pre-eclampsia from mild to severe can be rapid, unexpected, and occasionally fulminant. Maternal deaths can occur among the most severe cases. Despite intensive research for the past decades, the pathogenesis of preeclampsia has not been fully elucidated. A leading hypothesis (referred as the two-stage model) states that pre-eclampsia is initiated by disturbances in placentation at the beginning of pregnancy, followed by generalized inflammation and progressive endothelial damage (reviewed in [1,2,3]). However, pre-eclampsia is more likely a heterogeneous disorder [4]. Chronic hypertension, diabetes mellitus, obesity, nulliparity and adolescent pregnancy are associated with the risk of onset of pre-eclampsia. Studies have also shown that low levels of serum 25-hydroxyvitamin D in pregnancy are associated with pre-eclampsia [5,6], although there is currently no evidence supporting that supplementation of vitamin D lowers the risk on developing preeclampsia [7]. The first line of treatment includes α_2_-adrenergic receptor agonists, non-selective β-blockers, calcium channel blockers, and vasodilators to deal primarily with the signs [8]. Unfortunately, the only effective intervention is termination of pregnancy or delivery of the fetus and placenta.

The birth of an infant before 37 weeks of gestation is termed preterm birth. The obstetric precursors leading to preterm birth are: (1) delivery for maternal or fetal indications, in which labor is either induced or the infant is delivered by pre-labor caesarean section; (2) spontaneous preterm labor with intact membranes; and (3) preterm premature rupture of the membranes irrespective of whether delivery is vaginal or by caesarean section [9]. Preterm birth is associated with high risks of adverse neonatal sequelae, including cerebral palsy, language and learning disabilities, and poor growth due to the incomplete development of organs of the premature infants. Despite improvements in prenatal care, the number of preterm births has been rising. It was estimated that 12.9 million births, or 9.6% of all births worldwide, were preterm in 2005 [10]. The number of cases rose to 14.9 million, or 11.1% of all live births worldwide, in 2010 [11]. More than 60% of the cases occurred in South Asia and sub-Saharan Africa [11]. Furthermore, according to the more recent figures from the World Health Organization (WHO), preterm birth complications are thought to be responsible for nearly 1 million deaths in 2013 globally [12]. The situation is especially dire in low- and middle-income countries where 98% of all neonatal deaths occur [11]. The causes of preterm birth are multifactorial and complex. However, the most common associations with preterm birth include multiple pregnancies, intra-amniotic infections, and chronic conditions, such as diabetes mellitus and hypertension. There is also a genetic influence. However, the pathogenesis of preterm labor is not well understood and few of the proposed biomarkers meet the criteria to be considered clinically useful for predicting preterm birth [13]. It is thought that preterm labor might represent early idiopathic activation of the normal labor process or the results of pathological insults [9]. Recent studies have also linked vitamin D deficiency with the likelihood of preterm birth and neonatal outcomes [14,15].

Mass spectrometry (MS) is an indispensable analytical technology to proteomics and metabolomics. The technology has been employed in research and clinical settings to examine different diseases and conditions, including discovery of protein and metabolite biomarker of gestational diseases [16,17,18], and screen of inborn error of metabolism [19]. However, for the majority of the studies in reproductive medicine, classical biochemical assays, such as Western blot, enzyme-linked immunosorbent assay (ELISA), immunostaining, and real-time polymerase chain reaction (RT-PCR) remain the preferred methods. The design of these studies has also been hypothesis-driven and derived from pathways implicated in the pathogenesis of the conditions under investigation. While they have produced useful information and insight, the hypothesis-driven approach alone has not bought us close to answer the questions as to the pathogenesis or biomolecular mechanism of a diverse disease such as preeclampsia. Researchers conducting clinical science do not often have knowledge and expertise in MS technologies and have rarely taken advantage of the techniques, despite the increasing familiarity with systems biology approaches and hypothesis-generating strategies. This work describes and evaluates the advantages, limitations, and the recent advances of the MS methodologies that have been successfully applied in pregnancy-related complications. Emphasis is placed on the studies of pre-eclampsia and preterm birth.

## 2. An Overview of Proteomic Technologies

The proteome is the entire complement of proteins that is or can be expressed by a cell, tissue, or organism. The study of proteomes and their functions is referred as proteomics. The proteome is highly complex and dynamic since protein expression and turnover vary with cell type and the physiological state of the cells. In addition, protein transformations occurring through post-translational modifications (PTMs) (e.g., phosphorylation, glycosylation acetylation and methylation), and chemical damages (e.g., glycation, oxidation, and nitration) further increase the diversity and heterogeneity of the proteome. Furthermore, the dynamic range in expression of proteins in a biological system is normally several orders in magnitude. The interested proteins are frequently of low concentration and their activities are masked by high abundant proteins present in the system. Separation technologies for protein or peptide fractionation, depletion and enrichment are essential to reduce sample complexity [20]. A range of commercial systems is now available for immunodepletion including Agilent Technologies’ Multiple Affinity Removal System (MARS) series of columns, and SigmaAldrich’s ProteoPrep20. That said, other proteins are concomitantly removed during immunodepletion processes due to non-specific binding to the depleted proteins. Systematic analysis has proven these methods increase protein identifications and the representation of membrane and intracellular proteins analyzed by MS [21]. Even with the extensive range of techniques available, the proteome has proven difficult to analyze.

Mass spectrometric methods allow researchers to acquire mass spectral profiles of biofluids, cells or tissue samples. A series of mass spectra captures a snapshot of the proteome (or the metabolome) of a biological system under a specific condition, and ideally, each of the mass spectral features (a set of peaks in the mass spectrum) represents a biomolecule: a protein, a peptide or a metabolite. The change in concentration or differential expression of those biomolecules between the case and the control groups may indicate a discriminatory or diagnostic factor of the disease under investigation, or may inform as to the pathophysiology. These discriminatory biomolecules have often been mislabeled as “biomarkers”, when they should be called “biomarker candidates” [22]. These candidate biomarkers should then be verified and validated independently, by different analytical techniques, and tested for their sensitivity and specificity in a wider population. Very often one biomarker does not have the specificity to distinguish one condition from another, and hence a set of several biomarkers is often necessary as a determinant. A suitable analytical technique for biomarker discovery should be able to detect all these potential biomarkers indiscriminately, quantitatively and simultaneously in a single experiment, and be able to handle a large amount of samples in a relatively short time (*i.e.*, high-throughput).

**Figure 1:**
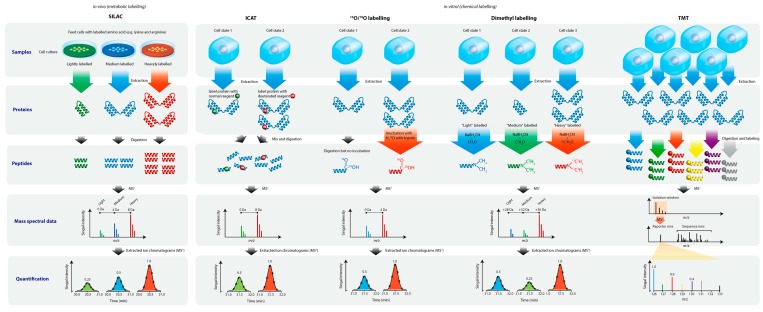
Commonly used label-based semi-quantitative proteomic methods. Two mains approaches are employed. Isotopic labels are incorporated into proteins or peptides by biochemical of the cells using labelled lysine and/or arginine, or by chemical reactions with labelling reagents. Quantitative data can be acquired via MS^1^ measurement, where the mass different between unlabeled and labeled peptides must at least be 4 Da. Alternative methods use (4- to 10-plex) isobaric mass tags to chemically label tryptic peptides from groups of sample. The tags contain four regions, a mass reporter region, a cleavable linker region, a mass normalization region and a protein reactive group. Upon fragmentation of the isobaric peptides by CID or HCD, fragmentation of the tags gives rise to mass reporter ions for quantification. The *m*/*z* values of reporter ion have been specifically designed at mass region away from typically interferences. Sequence information of the peptide back bone is also obtained simultaneously. The fragment energy is typically set slightly higher to promote complete fragmentation of the tags.

Studies of the proteome are classified as top-down (the analysis of intact proteins and peptides) and bottom-up approaches (the analysis of proteolytic peptides that surrogate the proteins of interest). The latter can be further categorized as discovery-based/shotgun (untargeted analysis) and targeted proteomics (targeted analysis) [23]. Quantitative proteomic analyses by shotgun proteomics have been typically performed with label-based methods [24,25,26,27], although label-free methods are gaining popularity [28,29]. The label-based methods achieve differential analysis by tagging proteins or peptides with light or heavy isotopes. These labelling methods were performed either *in vivo*, for example, by stable isotope labelling by amino acids in cell culture (SILAC) [30,31], or *in vitro*, by methods including dimethyl- [32,33] and ^16^O/^18^O-labelling [34], isotope-coded affinity tag (ICAT) [35,36], isobaric tags for relative and absolute quantification (iTRAQ) [37] and tandem mass tags (TMT) [38] (Figure 1). *In-vivo* techniques such as SILAC have higher quantification accuracy than *in vitro* approaches, notwithstanding that applications are limited to cultured cells. These isotopic labelling methods are also considered as MS1- (SILAC, ICAT, dimethyl- and ^18^O/^16^O-labeling), or MS2-based quantification (iTRAQ and TMT) [39]. iTRAQ and TMT differ from other labelling methods since they are multiplex approaches that use four, six, or eight isobaric tags to label tryptic peptides. The tagged peptides from subdivisions in a set therefore have an identical molecular weight, but produce reporter ions of different *m*/*z* during MS2 fragmentation. Furthermore, iTRAQ and TMT analysis can be performed either on a laser mass spectrometer coupled to off-line liquid chromatography (LC) separation, or by electrospray ionization on a Orbitrap, quadrupole time-of-flight (Q-ToF) or quadrupole linear ion-trap (LTQ) mass spectrometer coupled to online LC separation. Major limitations of iTRAQ and TMT are their relatively poor linearity and quantification accuracy [40,41], consequent to the use of reporter ions for quantification and the issue of ratio suppression (an underestimation of the relative change due to co-fragmentation of co-eluting ions with similar *m*/*z*). Two advanced methods that seek to overcome the problem of ratio suppression have been developed on the Orbitrap platform [42]. One method is based on a second stage of fragmentation (MS3) to eliminate interference [43], and another uses proton-transfer ion-ion reaction to generate purified precursors for further fragmentation [44]. However, many published iTRAQ works have been performed on unsophisticated Q-ToF (e.g., QStar) systems. The inaccuracy of differential changes and omission of low abundant proteins is likely high. Interpretation or construction of a biochemical model of a complex disease based on iTRAQ LC-Q-ToF methods may not bear any biological significance, before any changes in protein expression can be verified and validated (see later). Nevertheless, the power of shotgun proteomics is not to determine candidate biomarkers, but to narrow down the search of thousands of proteins potentially present in a biological system to around 50 or less proteins for further investigation by targeted proteomics [45] and/or biochemical assays [46].

Targeted proteomics has grown in popularity recently and the technique has been recognized by the journal Nature Methods as the method of the year 2012 [47,48,49]. Targeted proteomics uses a specific tandem mass spectrometry data acquisition mode, termed, selected reaction monitoring (SRM), in which the selected transition of an ion before and after fragmentation is measured. The method has the advantages of being sensitive and specific, and thus has the characteristics required to verify the data acquired by shotgun proteomics. SRM experiments are typically performed on a triple quadrupole mass spectrometer. Recent technological advances in targeted proteomics have also driven the introduction of data independent analysis and hyper reaction monitoring that “simulate” SRM measurements using high resolution fragmentation mass spectra acquired by a fast-scanning Q-ToF or Orbitrap system [50]. To obtain absolute quantification by LC-SRM, isotopically labelled references (e.g., synthetic peptides [51] or proteins [52]) are required. However, the difficulty and the relatively high cost associated with the synthesis and preparation of purified stable isotope encoded peptides or proteins have made the use of isotopically labelled references unattractive. One interesting approach utilizes recombinant DNA techniques and a rapid growing microorganism (*Escherichia coli*) to generate a library of stable isotope-labelled artificial proteins (QconCATs) [53,54]. The labelled QconCAT proteins can then be purified, quantified and added to complex protein mixtures in known amounts as labelled internal references to circumvent many of the problems in using synthetic peptides or proteins for absolute quantification. On the other hand, some studies have adopted a “label-free” approach to perform targeted relative quantitation by LC-SRM. A limitation of the label-free SRM approach is that it does not readily allow comparison of data sets from samples run at a different mass spectrometer or even the same system at different times.

## 3. Top-Down Proteomic Profiling by Laser Mass Spectrometry

**Figure 2:**
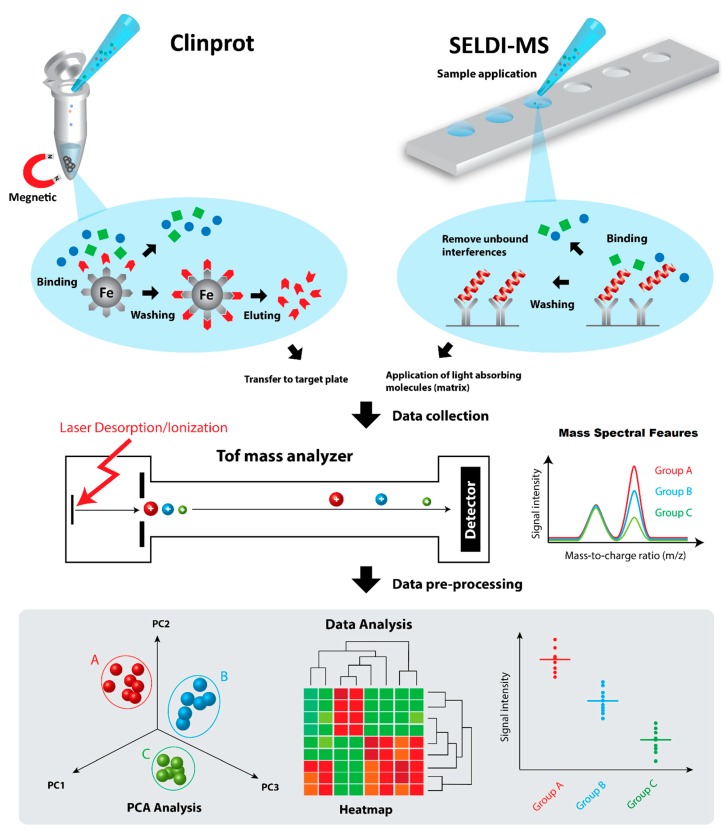
SELDI and Clinprot protein marker discovery workflow. Complex biological fluid, such as serum, urine, cerebrospinal fluid, cell and tissue lysates, is applied directly on SELDI ProteinChip or mixed with Clinprot magnetic beads. After incubation, unbound proteins and other contaminants, such as salt and detergent, are washed off. Interested proteins/peptides are captured onto their surface. The purified proteins/peptides are then analyzed directly by MALDI-ToF-MS. The mass spectral profiles are then analyzed by univariate or multivariate statistical data analysis for pattern recognition or classification.

Clinical top-down proteomics is conventionally conducted by two-dimensional (2D) differential gel-based electrophoresis analyses (e.g., 2D-PAGE, 2D-DIGE) [55]. The role of MS was often limited to protein identification. Biological samples such as blood plasma [56], serum [57], placental tissues [58,59,60,61], amnion and/or amniotic fluid (AF) [62,63] have been investigated for pre-eclampsia and preterm birth. However, 2D gel electrophoresis is limited in sensitivity and can be inefficient when analyzing hydrophobic proteins or those with very high or low molecular weight. In contrast, laser mass spectrometry meets all the aforementioned criteria for biomarker candidate discovery. Both matrix-assisted laser desorption/ionization mass spectrometry (MALDI-MS), and its variant, surface-enhanced laser desorption/ionization mass spectrometry (SELDI-MS), are now standard methods in protein/peptide biomarkers discovery in clinical settings [64] (Figure 2). SELDI-MS has been applied to acquire proteomic profiles of cerebrospinal fluid [65], amniotic fluid [66,67,68,69,70,71], and urine [72,73,74] to determine protein biomarkers for pre-eclampsia and intra-amniotic infection (IAI)—a major cause of preterm birth. Serum proteomic profiles obtained by MALDI-MS have also been used to differentiate pre-eclampsia patient samples from those of healthy controls [75,76]. Various potential protein biomarkers for pre-eclampsia and IAI were identified (Table 1). The biofluids are not analyzed directly by SELDI-MS or MALDI-MS. Biological matrices often contain low concentration of proteins, but relatively high concentration of electrolytes and other interfering substances, which often lead to ion suppression and/or spectral interferences. Intact proteins are extracted, desalted, and enriched from the matrices prior to proteomic profiling. Work-up procedures were conducted on the ProteinChip system in SELDI-MS, or ClinProt purification system in MALDI-MS. Both the SELDI ProteinChip (Bio-Rad Laboratories) and ClinProt (Bruker Daltonics) technologies explore the selective interactions or affinities of proteins to a number of selected sorbents, including hydrophobic reversed-phase, ion exchange, immobilized metal affinity capture (IMAC), and immunoaffinity substrates [77,78]. The differences between the two technologies largely lie on the former being a chip-based approach (*i.e.*, the sorbent is made on the surfaces, sample wells, of ProteinChip), and the latter being a magnetic bead-based method (*i.e.*, the sorbent is made on the surface of iron core particles). A wide selection of sorbent materials allows optimization for maximum retention of the interested proteins from the sample solutions. After incubation and washing steps, light-absorbing organic matrix is then applied onto the surface of the ProteinChip, or is co-crystallized with the proteins purified by ClinProt particles on a MALDI target plate. The MS profiles of the retained proteins are then acquired on a ToF mass spectrometer. ToF has a theoretically unlimited upper mass detection limit that makes SELDI-ToF-MS and MALDI-ToF-MS the methods of choice for intact macromolecule analysis. A limitation of the approach is that proteins biomarker candidates cannot be identified directly prior to their digestion to peptides. However, peptides of interest (up to 4 kDa) can still be sequenced in a ToF/ToF system.

**Table 1:** The Applications of MALDI-MS and SELDI-MS for Preeclampsia and Preterm Birth.

Technique	Sample	Cohort	Sample Preparation	Results	Biological Implications	Ref.
MALDI-MS(ClinProt)	Serum	Early-onset sPE: 11 CRL: 13	Sample extraction and enrichment was performed on HIC8 reverse phase coated magnetic beads. 5 μL of serum was incubated with 10 μL MB-HIC8 binding buffer and 5 μL of MB-HIC8 bead slurry for 1 min. After washing twice with 100 μL of wash buffer, proteins were eluted with 10 μL of elution buffer. After MB-HIC8 extraction, protein solutions, 0.5 μL of protein solution was spotted directly onto a stainless steel target plate before matrix solution was added.	The best differentiating signals between the two sample groups were found at *m*/*z* 13,715, 13,834, and 13,891. The normalized intensities of these ion signals were on average lower in the PE group than in the control group. The ion signals were believed to belong to the protein transthyretin and its modified forms. Results were consistent with that obtained with SDS-PAGE analysis.	The reduction of transthyretin concentrations is expected during pregnancy due to plasma volume expansion. Transthyretin is synthesized by the liver and also secreted by placental trophoblasts where it binds extracellular T4, which in turn result in an increased internalization of the transthyretin-T4 complex. It has been suggested that transthyretin plays an important role in the transfer of maternal thyroid hormone to the fetal circulation, which could have important implications for fetal development.	[75]
SELDI-MS (WCX2 array)	Cerebrospinal fluid (CSF)	sPE: 7 mPE: 8 CRL: 8	Dry on-chip protocol: 5-μL of undiluted pooled CSF was dried onto individual spots.	A cluster of 4 peaks was observed in the 15–16.3 kDa region only from the CSF of the patients. These peaks were assigned to α- and β-chains of hemoglobin, and their glycosylated formed. The presence of hemoglobin in CSF as biomarkers was validated with ELISA and spectrophotometry.	The reason for the observations was not clear. The authors suggested that the increase in CSF hemoglobin might result from increased and selective trafficking of intact erythrocytes across the blood-brain barrier where they subsequently lyse releasing their hemoglobin content.	[65]
SELDI-MS (Q10 array)	Amniotic fluid (AF)	PE: 18 CHTN: 7 CRL: 16	Before sample loading, each ProteinChip spot was incubated twice with 5 µL binding buffer in a humidity chamber at room temperature for 5 min. After equilibration, 5 µL of sample, diluted 1:3 in binding buffer, was added to each spot and incubated in a humidity chamber with shaking for 40 min. Each spot was washed with 5 µL binding buffer for 2 min, followed by washing with 5 µL of triple distilled water, and air-dried.	2 peaks located at 17,399.1 and 28,023.3 Da were significantly different. The former peak distinguished women with PE from control, and the latter peak distinguished women with PE and CHTN from controls. The peaks were assigned to hypothetical protein SBBI42 and proapolipoprotein A-I. The results were cross-validated with Western blot.	It was suggested that the increase in levels of proapolipoprotein A-I in the AF of women with PE may represent a compensatory mechanism to maintain levels of apolipoprotein A-I and thereby pulmonary surfactant and lung compliance and development.	[70]
SELDI-MS (H50 array)	Amniotic fluid (AF)	PE: 10 CRL:10	AF was obtained by transabdominal amniocentesis. Protein chip arrays were placed in a bioprocessor and pre-treated with 50% methanol for 5 min. 2 μL of AF and 3 μL of protein buffer were placed on individual sample spots and incubated at room temperature for 30 min. Each sample spot was equilibrated by adding 200 μL of binding buffer. After shaking for 5 min at room temperature, the buffer was removed, then, 5 μL of sample mixture and 195 μL of binding buffer were added to each spot and incubated with vigorous shaking for 30 min. The arrays were washed with 3 times of 200 μL binding buffer, followed by 2 times of 200 μL distilled water. The protein chip arrays were then air-dried.	5 protein peaks located at *m*/*z* 4679, 9080, 14,045, 14,345 and 28,087 were significantly differentially expressed between case and control groups. The peak located at *m*/*z* 14,345 was characterized as fragmented albumin, whereas the peak located at *m*/*z* 28,087 was identified as apolipoprotein A-I. The increased expression of apolipoprotein A-I was confirmed with ELISA.	The reason why albumin fragment may be overexpressed in these women is not immediately apparent. One possibility is that the increased proteolytic activity against albumin is an early phenomenon that precedes the clinical manifestations of the disease. Apolipoprotein A-I is expressed in the placenta and acts as a receptor for cholesterol, which is then transferred to the fetus. The origin of apolipoprotein A-I within AF remains unclear. Even less well understood is why apolipoprotein A-I is overexpressed in second trimester AF of women destined to develop pre-eclampsia. Whether the increase in apolipoprotein A-I in the AF precedes the increase seen in maternal plasma and urine in women with pre-eclampsia is not known.	[71]
SELDI-MS (Q10 array)	Urine	sPE: 11 mPE: 7 CRL: 8	30 μL aliquots of individual urine samples were mixed with 10 μL of sample buffer. Following 30 min incubation at room temperature, 160 μL of binding buffer was added to each sample. After equilibration of the ProteinChip Array, 150 μL of diluted sample mixture was loaded onto each spot and incubated with vigorous shaking for 1 h. Each spot was washed and air-dried.	4 discriminatory protein peaks were identified at *m*/*z* 4155, 6044, 6663 and 7971. All of these proteins had a lower concentration in urine. The identities of the protein biomarkers were not assigned, but their discriminatory power was tested with ROC.		[72]
SELDI-MS (H4 and H50 array)	Urine	sPE: 38 CRL: 21	ProteinChip arrays were incubated for 1 h with the samples (6 μL/spot) diluted to 0.25 mg/mL total protein. Following incubation, unbound proteins were removed by washing each spot with the respective buffer and dried.	At the end of exploratory phase, urine proteomic profiles from the patients with sPE exhibited 13 peaks qualitatively different from that of the controls. These peaks were assigned to non-random cleavage products of serpin peptidase inhibitor-1 (SERPINA1) and albumin protein. Urine proteomic profile score was tested against a cross-sectional cohort (*n* = 206). Performance was evaluated by ROC (AUC = 0.92). The over-expression of SERPINA1 in urine was also verified with western blot.	Other studies have shown that minor increases in levels of serum SERPINA1 are associated with the development of arterial hypertension and an increased risk of cardiovascular disease. The authors suggested that by inhibiting the activity of the kallikrein-kinin system, an up-regulation of plasma SERPINA1 favors the renin-angiotensin system, leading to systemic vasoconstriction and hypertension. Urinary albumin excretion is a hallmark of PE.	[74]
SELDI-MS (NP20, H4 and IMAC array)	Amniotic fluid (AF)	Preterm +IAI: 11 Preterm only: 11 CRL: 11	0.5 to 3.0 µg of unfractionated protein from AF was deposited on 3 different ProteinChip arrays (normal-phase SiO_2_, a reverse-phase hydrophobic, and immobilized nickel surfaces). The Chips were incubated for 1 hour with the sample followed by a 5-µL water wash, and subsequently dried.	A set of peaks located at 10- to 12-kDa was differentially expressed. The peaks were observed on all 11 patients with subclinical IAI, in 2 of 11 with preterm delivery without IAI, and in 0 of 11 with preterm labor and term delivery without infection. The signatures were identified polypeptides derived from calgranulin B and a unique fragment of insulin-like growth factor binding protein 1 (IGFBP-1). Results were validated by western blot.	The calgranulins are members of the S-100 calcium-binding protein family, expressed by macrophages and by epithelial cells in acutely inflamed tissues. The second candidate from this cluster, a specific proteolytic fragment of IGFBP-1, indicates a potential protease-related mechanism in response to infection. Intact IGFBP-1 is the major IGFBP found in AF and is synthesized by both fetal membranes and maternal decidua.	[66]
SELDI-MS (H4 array)	Amniotic fluid (AF)	Preterm (+WBC; +AFC): 21 Preterm (+WBC; −AFC): 7 Preterm (−WBC; +AFC): 8 Preterm (−WBC; −AFC): 24 CRL: 17	2 μL of AF diluted 10-fold in PBS. After 1-h incubation in a humidified box, the sample was aspirated and the spots washed individually with 25% aqueous acetonitrile solution, air-dried.	Candidate makers were tested on a separate set of 24 samples by blinding independent examiners to the outcomes. 3 additional samples were used to assess the possibility of storage artefacts and to calculate intra- and inter-rater agreement among the 3 investigators. 4 proteins, neutrophil defensins-1 and -2, and calgranulins A and C were found distinctive and were validated with western blot.	Neutrophil defensins (α-defensins) belong to a family of cationic antimicrobial peptides. These key components of the host-defense mechanism exert their bactericidal activity by punching pores into bacterial membranes.	[67]
SELDI-MS (RS100)	Amniotic fluid (AF)	Preterm +IAI: 86 Preterm only: 86 CRL: 86	5 μL of anti-IGFBP-1 antibody or control IgG solutions was loaded onto the spot of pre-activated ProteinChip arrays and covalently coupled for 2 h at room temperature in a humidity chamber. Remaining reactive groups were blocked for 1 h with 2 mg/mL BSA in 50 mM Tris-HCl. The spots were washed 3 times with 10 μL of PBS. Then, 5 μL AF samples were loaded on the antibody-coated arrays and incubated for 1 h in the humidity chamber. Arrays were washed three times with PBS and rinsed once with water before air-drying.	The ProteinChip array-based immunoassay using SELDI showed that IGFBP-1 was largely in a full-length form in the AF of the patients with preterm labor without IAI, but significantly degraded in the AF pool of the patients who delivered preterm with IAI. This indicated a preferential production of IGFBP-1 fragments in the amniotic fluid of patients with IAI.	Consistent with the previous study that the proteolytic degradation of IGFBP-1 by matrix metalloproteinases (MMPs) and different MMPs generated fragments of IGFBP-1 of different masses.	[68]
SELDI-MS (CM10 and H50)	Amniotic fluid (AF)	Preterm +IAI: 60 CRL: 59	AF from each patient was diluted in sterile PBS at a 1:10 dilution and was added onto the ProteinChip.	39 peaks were distinguishing patients with preterm labor with IAI from those with preterm lab our but subsequently delivered at term. The study did not seek to identify these mass spectrometric features.		[69]

Q10: strong anion exchange; CM10/WCX: weak cation exchange; H: hydrophobic (reverse-phase); CRL: controls; PE: preeclampsia; sPE: severe preeclampsia; mPE: mild preeclampsia; CHTN: chronic hypertension; +IAI: intra-amniotic infection; +AFC: positive amniotic fluid culture results; +WBC: white blood cell count > 100 cells/mm^3^; PBS: phosphate buffered saline; BSA: bovine serum albumin.

Laser mass spectrometry used to have a relatively large technical variation (*i.e.*, low precision of measurement), such that the data acquired by the methodology was treated qualitatively. The technological advances of the MALDI technology, such as the use of high repetition rate solid-state laser, and improved instrument design, have increased the precision of measurement to less than 20% (demonstrated in a study using CLINPROT approach) [79]; this permits semi-quantitative measurement on biological systems by MALDI-MS.

When the two techniques are compared, the chip-based SELDI-MS approach has the advantage of being convenient. Relatively, the magnetic bead-based approach provides a greater surface area for the protein extraction, purification, and enrichment, although additional elution and sample deposition steps are required. As demonstrated in a study of urine from healthy volunteers, the number of mass spectral features (peptide peaks) detected were at least two times higher on ClinProt than on SELDI ProteinChip [80]. It was apparent that the improved sensitivity was due to the higher adsorption capability of the beads’ porous surface structure compared with on-layer chips. On the other hand, MALDI-based analysis has been enhanced through technical advances to the informatics used to conduct pattern discovery and data classification [69,81]. Bio-Rad ProteinChip data manager and Pattern Analysis Software offer functionality and statistical procedures that biomedical professionals are familiar with, such as receiver operating characteristic (ROC) analysis, principal component analysis (PCA), hierarchical clustering and classification and regression trees (CART) [82,83]. Bruker ClinProTools uses statistical methods such as modified Genetic Algorithm (GA), Support Vector Machine (SVM) and Quick Classifier (QC) for data analysis [77]. Both of these software systems perform data pre-processing, (baseline subtraction, spectral data realignment with prominent internal peaks, normalization of all spectra to their total ion count (TIC), and ion peak deconvolution) before conducting sophisticated statistical analysis [69,77,84].

## 4. Top-Down Peptidomic Profiling by Liquid Chromatography and Capillary Electrophoresis Mass Spectrometry

Naturally occurring peptides arising from the proteolytic cleavage of proteins could have biological activities that are different or even opposite to those of the parent protein [85]. Spectral profiles of the naturally occurring peptides (peptidomic profiling) can be acquired by coupling mass spectrometry to liquid chromatography (LC) or capillary electrophoresis (CE). CE is a microscale separation technique based on differential mobility of charged species in an electrical field. CE offers higher separation efficiency relative to LC, and is thus highly suitable for separation of complex samples [86]. Coupling CE to MS would therefore provide a remarkable capability to peptides separation [87]. One major limitation of CE-MS relative to LC-MS is that the loading capacity of CE does not permit peptide sequencing.

By using LC-MS, Wen, *et al.* were able to identify 612 serum peptides from normal pregnancies and form women with pre-eclampsia [88]. The peptidomic profiles were then analyzed by significance analysis of microarrays (SAM) and predictive analysis of microarrays (PAM). SAM identified 52 peptides derived from 14 protein precursors with highly significant differences in expression between pre-eclampsia and control samples. Nineteen unique peptides [13 from fibrinogen alpha (FGA), 1 from alpha-1-antitrypsin (A1AT), 1 from apolipoprotein L1 (APO-L1), one from inter-alpha-trypsin inhibitor heavy chain H4 (ITIH4), two from kininogen-1 (KNG1), and one from thymosin beta-4 (TMSB4), respectively] were selected for PAM prediction. On the training set, all pre-eclampsia samples (*n* = 21) were predicted correctly, while three of the 21 (14.3%) control samples were false positive. The sensitivity on the training set was 85.7% and the specificity was 100%, resulting in the overall prediction accuracy of 92.9%. Similarly, on the testing set, the overall prediction accuracy is 90%, with sensitivity 80% and specificity 100%. Pathway analysis (Ingenuity Pathway Analysis software) was performed on the 14 parental proteins of the 52-peptide markers. Liver X receptor (LXR)/retinoid X receptor (RXR) activation, atherosclerosis signaling, IL-12 signaling and production in macrophages, clathrin-mediated endocytosis signaling, production of nitric oxide and reactive oxygen species in macrophages, acute phase response signaling, coagulation system, farnesoid X receptor (FXR)/RXR activation and intrinsic prothrombin activation pathway are statistically significant canonical pathways that were implicated in the pathophysiology of pre-eclampsia. Alternatively, by using CE-MS, Carty, *et al.* were able to identify a urinary peptide pattern at gestational week 28 from non-pregnant women, healthy pregnant, and those who had developed pre-eclampsia [89]. The peptides were characterized by breakdown products of fibrinogen, collagen, and uromodulin (by LC-MS), which could differentiate women who subsequently develop pre-eclampsia.

## 5. Bottom-Up Quantitative Proteomics

### 5.1. Serum and Plasma

The international SCreening fOr Pregnancy Endpoints (SCOPE) study used a combination of shotgun and targeted proteomics and a biochemical assay (ELISA) to screen and pinpoint the plasma protein biomarkers for predicting pre-eclampsia [90,91]. During the analytical pipeline development [90], the researchers utilized an iTRAQ approach to screen early pregnancy plasma samples from women who subsequently developed pre-eclampsia. The pooled plasma was first immunodepleted using either a SepproÏgY 14-SuperMix Liquid Chromatography Column system, or Agilent Multiple Affinity Removal LC Column—Human 14 (MARS Hu-14) to remove the most abundant proteins in the plasma. The immunodepleted samples were then digested and labelled with 8-plex iTRAQ reagent. The labelled samples were fractionated using high pH reversed-phase chromatography, and the fractions were then analyzed either by online reversed-phase nano-LC coupled to a QStar XL qTOF system, or by a MALDI TOF-TOF after off-line LC separation. 502 proteins were identified across 3 iTRAQ discovery experiments (319 proteins in the IgY 14-SuperMix- QSTAR data set, 331 in the IgY 14-SuperMix5800 data set and 189 in the MARS 14–5800 data set) and a total of 113 proteins altered in abundance. Following application of stringent candidate protein selection criteria, two proteins: pregnancy-specific beta-1-glycoprotein 9 (PSG9) and platelet basic protein (PBP, CXCL7), were subjected to further assessment by SRM-based verification. In the targeted analysis phase, 108 plasma samples, which include 16 early-onset preeclampsia (EO-PE), 42 late-onset preeclampsia (LO-PE), 42 controls, and eight technical replicates of a pooled sample, were immunodepleted on MARS Hu-14 immunodepletion column. The depleted plasma was then fractionated on an mRP-C18 column prior to tryptic digestion. The tryptic peptides were then analyzed by LC-SRM on an uHPLC QqQ system. The authors observed that six PSG specific peptides (from PSG-9 and PSG-5) were significantly different between EO-PE and controls. The results were verified with ROC analysis and ELISA-based quantification. On the other hand, only one of the PBP peptides measured was found significant. Nevertheless, the authors noted that iTRAQ-based quantification has only a modest proteome penetrance [90]. In their further study [91], the plasma proteins were tagged with ^16^O/^18^O-labelling method. The samples were first depleted of the most abundant proteins using MARS Hu-14 columns. Plasma proteins were then acetylated with sulfo-*N*-hydroxysuccinimide acetate prior to tryptic digestion. ^18^O-label from H_2_^18^O was incorporated into the peptides after incubation at 37 °C for 26 h with trypsin. Furthermore, *N*-terminomics COFRADIC (COmbined FRActional DIagonal Chromatography) platform was adopted from [92] to reduce the sample complexity prior to off-line LC separation and MALDI-ToF/ToF analysis. Sixty-four proteins found in the discovery phase were chosen for verification, including a number of reported markers for pre-eclampsia, such as endoglin [93], disintegrin and metalloproteinase domain-containing protein 12 [94]. Additionally, nine proteins previously identified in a cardiovascular biomarker study with biology relevant to pre-eclampsia and three proteins (placental growth factor, soluble fms-like tyrosine kinase-1, and placental protein 13) with a recognized association with pre-eclampsia were also taken forward to the verification experiments. SRM assays were successfully developed for 51 out of the 76 selected proteins. Forty-four models passed the verification stage, of which eight reached the target performance of 50% sensitivity at 20% positive predictive value (PPV) for 5% prevalence in the validation set. These eight validated models composed of mean arterial pressure (MAP) and 11 combinations of the proteins:

- insulin-like growth factor acid labile subunit (IGFALS),
- endoglin (ENG),
- disintegrin and metalloproteinase domain-containing protein 12 (ADAM12),
- serine peptidase inhibitor Kunitz type 1 (SPINT1),
- melanoma cell adhesion molecule (MCAM),
- selenoprotein P,
- multimerin-2,
- extracellular matrix protein 1,
- microtubule-associated protein RP/EB family member 1 or 3,
- fructose-bisphosphate aldolase A,
- placental growth factor (PlGF)

The eight validated models all showed very similar performance for overall pre-eclampsia prediction, but IGFALS carried the most predictive weight [91].

Similar approaches have also been adopted in independent investigations to determine serum biomarkers for preeclampsia [95,96,97], albeit the scales of the cohorts were relatively small when compared to the SCOPE study. In one study [95], the six most abundant proteins from the serum samples were also first depleted with MARS, but the relative expression of the identified proteins was compared by a label-free approach based on spectral counting. Four protein candidates, α-2-HS-glycoprotein (AHSG), insulin-like growth factor binding protein, acid labile subunit (IGFBP-ALS), retinol binding protein4 (RBP4) and alpha-1-microglobulin/bikunin (AMBP), were selected for further SRM assay. The expression of IGFBP-ALS, AMBP and AHSG was higher in serum of the pre-eclampsia group, whereas the expression of RBP4 was reduced when compared to the normal group. The increased AHSG expression in serum was validated with ELISA [95].

In another study [96], peptide ligand library beads (PLLBs)-based affinity resin (ProteoMiner) was employed for capturing the low abundance proteins. The isolated proteins were then separated by SDS-gel and subjected in-gel digestion. LC-MS/MS analysis of the tryptic peptides was conducted on a LTQ-Orbitrap mass spectrometer coupled to a nano-LC system. Relative quantitation of protein expressions was achieved by spectral counting. Using two-fold or more changes as a determinant, it was found that 31 proteins were up-regulated and 20 were down-regulated in patients with pre-eclampsia comparing with healthy pregnant women. Among them, two proteins, chorionic somatomammotropin hormone (CSH) and fibulin-1, were of particular interest, since they are low abundant proteins and are seldom implicated in pre-eclampsia. The increased expression of apolipoprotein E (ApoE) in serum from women with pre-eclampsia was verified with MS1 data using an isotope-encoded peptide corresponding to a tryptic peptide of ApoE. Transthyretin (TTR), retinol-binding protein 4 (RBP4), CSH and α-2-HS-glycoprotein (AHSG) were chosen for further validation by Western blot. The immunoblot analysis showed that the concentrations of TTR and RBP4 were six and four times respectively lower in the serum of patients with severe pre-eclampsia than in that in controls. In contrast, the expression of CSH and AHSG were increased in severe pre-eclampsia. All differentially expressed proteins were then analyzed using DAVID Bioinformatics Resources to connect the proteins to biological processes and pathways. It was demonstrated that 17 of the differentially expressed proteins were linked to complement and coagulation response and 15 were acute phase response proteins. Six proteins were extracellular matrix proteins. The analysis also revealed three major clusters of molecular function: seven proteins were endopeptidase inhibitors, five were calcium-ion-binding proteins and eight were heparin-binding proteins. Collectively, these observations implicate that acute phase, complement and coagulation responses play a major role in pre-eclampsia [96].

### 5.2. Placental Tissue

While the SCOPE study aimed to determine circulating biomarkers for predictive or diagnostic purposes, other researchers have chosen to perform comparative proteome analyses on human placenta tissue between normal pregnancies and pregnancies complicated by pre-eclampsia, in order to determine the biological processes involved in the development of the condition [98,99]. Placental tissue was dissected from the maternal side of the placentas (in the central part, exclusive of calcified areas). After homogenization, extraction and digestion, tryptic peptides were dimethyl-labelled. LC-MS/MS analysis was performed on a LTQ-Orbitrap-Velos mass spectrometer coupled to a NanoACQUITY UPLC system and 2636 unique proteins were expressed in the human placental tissue. Kyoto Encyclopedia of Genes and Genomes (KEGG) pathway analyses were performed on the identified proteins to determine the relevant pathways. Most of the identified pathways were consistent with the development of placenta, including 297 proteins predicted to be involved in the metabolic pathways. Further examination revealed that 171 proteins were differentially expressed between normal pregnancy and pre-eclampsia; 147 proteins were down-regulated, while the remaining 24 proteins were up-regulated in pre-eclampsia. Endoglin (ENG), ceruloplasmin (CP), mitochondrial superoxide dismutase (SOD), transforming growth factor beta (TGF-β), are all thought to play key roles in the development of pre-eclampsia; each was differentially expressed. Gene ontology (GO) analysis implicated oxygen transport, regulation of cell death, and the aminoglycan metabolic and homeostatic processes in the condition [98].

The authors then performed a comparative proteome analyses to determine the *N*-glyco- and phospho-proteins that were differentially expressed in human placental plasma membrane [99]. Glycosylation and phosphorylation were chosen because these PTMs are known to be important in cell signaling, development, migration and recognition. The placental tissue was homogenized in plasma membrane isolation buffer. Enrichment and isolation of the plasma membrane was achieved by a two-layer step sucrose gradient. The glycans were released from glycopeptides using PNGase F. Phosphopeptides were enriched on Ti-doped mesoporous silica and 1027 *N*-glyco- and 2094 phospho- sites were identified in the human placenta plasma membrane. KEGG pathway analyses identified pathways related to the functions of the placental plasma membrane during pregnancy. Differences in glycosylation and in phosphorylation between the case and control groups were found in seven and 42 protein peptides respectively, corresponding to five *N*-glyco- and 38 phosphoproteins; further KEGG pathway analyses revealed differences in biological processes that included focal adhesion, shigellosis, and bacterial invasion of epithelial cells. Furthermore, GO pathway analysis implicated a number of biological processes as involved in pre-eclampsia, including negative regulation of DNA repair, sterol regulatory element binding protein import into nucleus, and positive regulation of response to interferon-gamma.

The authors further investigated the placental mitochondria proteome because mitochondrial dysfunction of the placenta occurs in pre-eclampsia [100]. After mitochondria isolation from the placenta tissue, mitochondrial proteins were precipitated with cold acetone and then re-suspended in dissolution buffer. After digestion, tryptic peptides were labelled with the iTRAQ regents, subsequently fractionated by strong cation-exchange (SCX) chromatography and analyzed by a LTQ-Orbitrap mass spectrometer coupled to a nano-LC system. 26 differentially expressed mitochondrial proteins were identified; 22 were down-regulated in pre-eclampsia, many of which are implicated in apoptosis, fatty acid oxidation, the respiratory chain, reactive oxygen species generation, the tricarboxylic acid cycle and oxidative stress. Due to the lack of suitable antibodies, only three of the proteins (transferrin receptor (TFRC), mitochondrial protein peroxiredoxin III (PRDX3) and heat shock 10kDa protein 1 (HSPE1)) were verified with Western blot and immunohistochemical analysis. However, the results were consistent with that of iTRAQ analysis [100].

### 5.3. Trophoblast Cells

The placenta predominantly consists of trophoblast cells, which have multiple functions, including adhesion and invasion during early pregnancy and an important secretory role in late pregnancy. Trophoblast cell isolation from placental tissue eliminates the problem of tissue heterogeneity of placental tissue.

In one of the studies, laser capture microdissection was used to isolate placental trophoblastic cells. Cellular proteins were extracted from the cellular lysates, and were labelled with ICAT reagents prior to tryptic digestion and LC-MS/MS analysis. 70 proteins (note: eight entries were duplicated) showed quantitative differences between pre-eclampsia and normal pregnancies (with a cut-off ratio of two); 31 were down-regulated in pre-eclampsia, while 39 were up-regulated. Significantly up-regulated proteins included Fraser syndrome 1 isoform 2, pancreatic tumor-related protein and KIAA0226 protein; down-regulated proteins included laminin (expression in trophoblastic cells was verified with Western blot) [101]. 

In another study, the proteins secreted by cultured cytotrophoblast cells from pre-eclampsia complicated pregnancies were analyzed using a label-free approach on a LTQ-Orbitrap-Velos coupled to a NanoAcquity system [102]. LC-MS/MS experiments were conducted on both pooled and individualized culture supernatants of cytotrophoblastic cells. From the analysis of individualized supernatant samples, 21 proteins were secreted significantly differently by cells from control and pre-eclampsia pregnancies. One protein was detected exclusively in supernatant of control cytotrophoblast cells and was identified as factor XIII chain A. This observation was further evaluated with RT-PCR analysis and mRNA expression of factor XIII chain A was significantly decreased in cytotrophoblastic cells from pre-eclampsia compared to the control.

### 5.4. Amniotic Fluid and Cervical-Vaginal Fluid

Clinical studies of AF and cervical-vaginal fluid (CVF) have been implicated in preterm labor and other pregnancy-related complications, such as Down syndrome and Turner syndrome (see ref. [16] for further discussion of the chemical constitutes of AF and CVF). In a study of the AF proteome, iTRAQ was employed to identify biomarker candidates of spontaneous preterm birth (sPTB) [103]. In a cross-sectional study by selecting AF samples from 75 patients with an episode of uterine contractions at preterm gestations and intact membranes. The cohort was classified into three groups: (A) preterm labor without intra-amniotic infection (IAI) but delivered at term; (B) preterm labor without IAI and delivered preterm within seven days of amnniocentesis; and (C) preterm labor with IAI. The AF samples used were obtained from transabdominal amniocenteses performed for evaluation of microbial status of the amniotic cavity. The samples were first depleted of albumin and Immunoglobulin G using Vivapure immunoaffinity anti-human serum albumin capture resins, and ultralink immobilized protein G. Acetone precipitation was also used to remove low molecular interferences. Individual samples were digested and labelled with 4-plex iTRAQ reagents (the three groups of samples were labelled with 115, 116, and 117 reagents and the pooled reference sample was labelled with 114 reagent). The combined labelled samples were fractionated by SCX (six fractions) and off-line LC separation before MALDI analysis. During the subsequent data analysis, the authors also excluded the CID spectra, in which the 114-reference reporter ion was not observed, from the determination of relative abundance of proteins. Overall, 77 proteins were up-regulated in preterm labor with IAI (group C), whereas six proteins were down-regulated. Seventy-nine of the differentially expressed proteins had been previously associated with infection/inflammation related preterm birth by hypothesis-driven research studies. GO analysis further revealed that these differentially expressed proteins have four main functions: (1) host defense; (2) mobility, localization and targeting; (3) anti-apoptosis; and (4) metabolism/catabolism. Fewer proteins were differentially expressed on comparison of preterm without IAI (group B), and delivered at term without IAI (group A). Four proteins (resistin (RETN), thymosin-like 3 (TMSL3), antileukoproteinase (SLPI), and growth-inhibiting protein 12 (lactoferrin, LTF)) were up-regulated, and two proteins (latent-transforming growth factor β-binding protein isoform 1L (LTBP1) and mimecan precursor (OGN)) were down-regulated. The altered AF resistin concentrations were verified with ELISA.

In a similar study [55], CVF was collected from subjects with sPTB, preterm labor without preterm delivery (PTL), and asymptomatic control subjects. Patients with IAI at time of presentation and/or placental histopathology at delivery were excluded. CVF proteins were analyzed by fluorescence 2D-DIGE and multidimensional LC-MS/MS analysis. For the LC-MS/MS analysis, individually pooled samples were prepared from five maternal CVF samples, each of control, PTL and sPTB. Non-protein interferences were removed by acetone precipitation. The tryptic peptides were separated into 80 fractions by SCX chromatography and each fraction was then analyzed on a Q-TOF-2 mass spectrometer coupled to a CapLC. Spectral counting was used to assess the relative abundance of a protein. Twenty-eight of the identified proteins exhibited significant differences in pairwise and progressive comparisons. Calgranulins A and B, annexins A3, S100 calcium-binding protein A7, and epidermal fatty acid binding protein were abundant in CVF and differentially present in PTL and sPTB samples, as were the serum proteins α-1-antitrypsin, α1-acid glycoprotein, haptoglobin precursor, serotransferrin precursor (transferrin), and vitamin D binding protein precursor [55].

Quantification of protein biomarker candidates by targeted proteomics is hampered by the difficulties associated with the preparation of labelled peptide references. One study took an opportunistic approach to generate an entire labelled proteome, which was then used as internal standards to facilitate accurate quantification of peptides, and proteins thereof, in a biological sample [104]. The method is termed stable isotope labeled proteome (SILAP). The SILAP method is based on the absolute SILAC approach [105], in which cultured cells are grown in stable isotope-labelled serum-free media with stable isotope labelled lysine and leucine. To demonstrate the technical capability of SILAP, columnar epithelial endocervical-1 (End1) and vaginal mucosal-2 (Vk2) cells were grown in SILAC conditions to generate a SILAP library containing the secreted proteins. It was assumed that End1 and Vk2 cells are “normal” transformed cells of human origin and have a secretory phenotype, and therefore, their secreted proteins would model the proteins present in human CVF. The secreted proteome of the cells were then determined by multidimensional LC-MS/MS analysis, after MARS Hu6 abundant proteins depletion and SCX fractionation. In three independent experiments (replicates), 1211 proteins were identified from the secreted proteome. Of the total 1211 proteins identified, 236 were detected in all three replicates. Fifteen potential protein biomarkers for sPTB were chosen from the 236 proteins based on the previous reports in sPTB or other pregnancy-related conditions. Stable isotope dilution LC-SRM assays were then designed to conduct relative quantification of these candidate biomarkers in term and sPTB human CVF samples spiked with the SILAP internal standard. Elevated expression of desmoplakin isoform 1, stratifin, and thrombospondin 1 precursor in sPTB relative to the control group was statistically significant. In particular, desmoplakin isoform 1 peptide was quantified to be 70.7-fold higher in the sPTB samples as compared to the control samples. Furthermore, stratifin peptide (42.4-fold) and thrombospondin 1 precursor peptide (5.1-fold) levels were significantly greater in the preterm birth patient samples. While the data indicated these three proteins could serve as potential protein biomarkers, the study was based on a relatively small cohort; further validation is required. 

## 6. Comments

### 6.1. The Current Status of Proteomics for Pre­Eclampsia and Preterm Birth

The technological advances of mass spectrometry and the associated techniques have provided a unique opportunity to examine pregnancy-related complications such as pre-eclampsia and preterm birth. A variety of proteomes has been examined by MALDI and SELDI-MS proteomic profiling. The applications of MS-based top-down proteomics have clear advantages over 2D gel electrophoresis, such as higher sensitivity to both hydrophobic and hydrophilic proteins. The use of a ToF mass analyzer also allows detection of very high mass proteins. Shankar *et al.* predicted 10 years ago that MALDI/SELDI-MS combined with pattern recognition would be essential to make use of the method for diagnostics [106]. The availability of informatic tools has now made this possible.

Various bottom-up quantitative or comparative proteomic studies have been conducted using a variety of label-based shotgun proteomic approaches. The iTRAQ technique has been by far the most popular approach employed, despite known limitations. Although numerous protein candidates have been discovered and reported, few of the proposed protein markers were actually validated. Only a small number of studies further employed targeted proteomics or biochemical assays to verify proposed biomarkers, and the applications of ELISA and Western blot were often limited by the availability of antibodies. LC-SRM-based assays are complementary to Western blot and/or immunohistochemical assays. Nevertheless, LC-SRM-based assays do not require suitable antibodies that are often commercially unavailable. High precision and accuracy would still require labelled reference peptides. Furthermore, some studies were based only on the data acquired from a relatively small cohort or case-control samples. The resulting models are at best dubious. In contrast, the SCOPE study established a powerful pipeline by combining both shotgun and targeted proteomics or biochemical assays, to ascertain the sensitivity and specificity of the proposed biomarkers for clinical diagnosis or probing the pathophysiological mechanisms. Other authors should note on the number of SRM transitions to be measured per peptide in a proper LC-SRM experiment. Measurements based on one SRM transition per peptide and/or one peptide per protein is less than ideal.

Meanwhile, challenges remain; the proteome is complex and many of the proteins of interest are of low abundance; 85% of human serum proteins by mass are comprised of six proteins. These high abundant proteins obscure potentially more biologically interesting low abundant proteins. Methods to deplete the high abundant proteins have been employed for isolating and enriching low abundant proteins in serum. A further issue concerns the variation in protein expression in different biological compartments. Non-invasive biological fluids such as serum and urine may be useful to identify diagnostic markers, but investigation of the pathogenesis of pre-eclampsia and other pregnancy-related complications may be best conducted using placenta tissue or trophoblast cells. AF and CVF samples may be particularly pertinent to the study of preterm birth.

### 6.2. Agreement between Observations of Studies and Their Possible Connections

**Figure 3:**
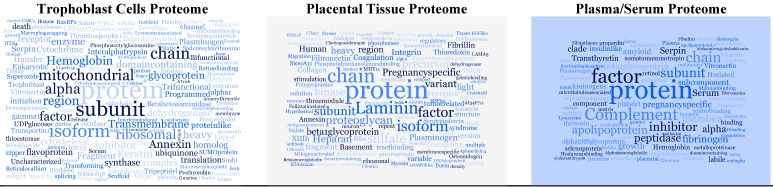
Most frequently reported differentially expressed proteins in the studies of pre­eclampsia. Cloud tags plots represent the number of proteins and frequency of a protein reported in a proteome.

Investigations on AFs by SELDI-MS have produced associated observations (Table 1). In contrast, studies conducted by discovery-based shotgun proteomics using data dependent analysis (DDA) produced disparate results. This can be contributed to the poor repeatability of DDA. Laboratories using different methods and MS systems obviously produce a diverging set of observations. We performed an analysis on the reported data to determine if there was any overlapping of observations and identified 13 differentially expressed proteins were reported more than once in all the MS-based proteomic studies for pre­eclampsia reviewed (Figure 3). These 13 proteins are involved in a variety of biological processes, but six of them are known to involve in cell adhesion and extracellular matrix organization. Plasminogen (or plasmin) was the only protein reported in serum, placental tissue, and trophoblast cells. Furthermore, different forms of apolipoprotein were reported in serum, placental tissue and AF for pre­eclampsia. Insulin-like growth factor and variants have also been reported not only in the studies of pre-eclampsia, but also in preterm birth. Histone H4 was found in AFs and CVF for preterm birth.

### 6.3. Clinical Assays of Biomarkers

The success of the SCOPE study has led to identification of a number of protein and metabolite markers and clinical risk factors for predicting pre-eclampsia in nulliparous women [91,107,108,109]. The results have been translated to clinical assays. A predictive test based on proteins identified through a LC-MS strategy is currently being validated through a phase IIa hospital-based study [110]. Further update can be seen at the IMPROvED web site (http://www.fp7-improved.eu/).

### 6.4. Future Directions

We anticipate that future investigations for pregnancy-related complications will take advantage of recent technological advances in MS. Novel fragmentation techniques such as electron transfer dissociation (ETD) may allow us to determine the protein identities or characteristics in top-down proteomic experiments. The method can further facilitate analysis of labile PTMs. The authors also recommend label-free approaches using data independent analysis (DIA), such as MS^E^ and all-ion-fragmentation, to enhance experimental repeatability. The recent introduction of Progenesis QI for proteomics has made DIA data processing much more accessible. Biomarker verification by targeted proteomics with conventional SRM and emerging hyper reaction monitoring (HRM), such as multiplexed DIA and parallel reaction monitoring performed on high resolution Q-ToF and Orbitrap systems, are expected to be more often reported in future studies. 

**Table:** 

Most Frequently Reported Proteins	Found in	Function
**alpha-2-HS-glycoprotein**	serum	Promotes endocytosis, influences the mineral phase of bone
**Ceruloplasmin**	serum, placental tissue	May play a role in fetal lung development or pulmonary antioxidant defense
**Endoglin**	serum, placental tissue	Crucial role in angiogenesis
**Fibrinogen alpha chain**	serum	Various cleavage products of fibrinogen and fibrin regulate cell adhesion and spreading
**Fibronectin**	serum, placental tissue	Cell adhesion and binding to various cell surfaces compounds
**Fibulin-1**	serum, placental tissue	Binds to a number of extracellular matrix constituents including fibrinogen and fibronectin
**Hemoglobin subunit alpha**	serum, placental tissue	Oxygen transport Embryonic hemoglobin in mammalian
**Hemoglobin subunit zeta**	serum, placental tissue	
**Plasminogen**	serum, placental tissue, trophoblast cells	Acts as a proteolytic factor in a variety of other processes including embryonic development, tissue remodeling
**Pregnancy-specific beta-1-glycoprotein 3**	serum, trophoblast cells	Secreted products of the syncytiotrophoblast; known to be extremely vital to development and health of a fetus
**Pregnancy-specific beta-1-glycoprotein 4**	serum, trophoblast cells	
**Transthyretin**	serum, placental tissue	Thyroid hormone-binding protein; thought to transport thyroxine from the bloodstream to the brain
**Vitronectin**	serum, placental tissue	Cell adhesion and spreading factor

## 7. Conclusions

We have chosen to discuss pre-eclampsia and preterm as two conditions that are the major causes of perinatal morbidity and mortality. Obstetricians face the challenge of predicting and diagnosing both conditions, particularly when women are in their first pregnancies, with no previous obstetric history. MS-based proteomics has been applied in various investigations of pregnancy-related complications. MS is a powerful alternative to conventional biochemical assays, but has not received adequate attention. The technology has been used to study biological fluids and cells cultures, and has identified various proteins that differentiate pre­eclampsia or preterm birth from healthy controls. Analysis of the scientific literature reveals that an extensive network of proteins has been reported to be significant. However, the overlapping of observations is relatively small on the same sample type or on different biological compartments. Many of the observations could be considered as different aspects of the disorders. Nevertheless, a pattern of biomarkers, for example, endoglin, α-2-HS-glycoprotein, fibrinogen, plasminogen, different apolipoproteins and insulin-like growth factor binding protein are found in various studies. Verifying these protein markers is essential for the clinical field to accept and use the results from marker discovery studies; a phase IIa hospital-based clinical study has already started. 
